# Effect of interventions including provision of personalised cancer risk information on accuracy of risk perception and psychological responses: A systematic review and meta-analysis

**DOI:** 10.1016/j.pec.2019.08.010

**Published:** 2020-01

**Authors:** Max Bayne, Madi Fairey, Barbora Silarova, Simon J. Griffin, Stephen J. Sharp, William M.P. Klein, Stephen Sutton, Juliet A. Usher-Smith

**Affiliations:** a University of Cambridge School of Clinical Medicine, Cambridge, UK; b MRC Epidemiology Unit, University of Cambridge, Cambridge UK; c The Primary Care Unit, Department of Public Health and Primary Care, University of Cambridge, Cambridge, UK; d Behavioral Research Program, National Cancer Institute, Rockville, MD, USA

**Keywords:** Cancer risk, Personalised risk provision, Systematic review, Intervention, Risk perception, Worry, Anxiety

## Abstract

**Objective:**

To synthesize the literature on the effect of provision of personalised cancer risk information to individuals at population level risk on accuracy of risk perception and psychological responses.

**Methods:**

A systematic review and random effects meta-analysis of articles published from 01/01/2000 to 01/07/2017.

**Results:**

We included 23 studies. Immediately after provision of risk information 87% of individuals were able to recall the absolute risk estimate. Less than half believed that to be their risk, with up to 71% believing their risk to be higher than the estimate. Provision of risk information increased accuracy of perceived absolute risk immediately after risk information compared with no information (pooled RR 4.16 (95%CI 1.28–13.49), 3 studies). There was no significant effect on comparative risk accuracy (pooled RR 1.39 (0.72–2.69), 2 studies) and either no change or a reduction in cancer worry, anxiety and fear.

**Conclusion:**

These findings highlight the complex cognitive processes involved in the conceptualisation of risk.

**Practice implications:**

Individuals who appear to understand and are able to recall risk information most likely do not believe it reflects their own risk.

## Highlights

• Conceptualisation of risk is a complex cognitive process.
• Individuals tend to overestimate their risk of cancer at baseline.
• Immediately after risk information over 80% of people are able to recall the number.
• However, less than half believe that to be their risk, thinking their risk is higher.
• Risk information has either no effect or reduces worry, anxiety and depression.

## 1 Introduction

An increasing number of risk models are now available that enable estimation of an individual’s future risk of cancer. Although providing individuals with a personalised risk estimate in isolation is unlikely to lead to behaviour change [1,2], personalised risk communication may complement educational interventions and increase motivation and health-related behaviour change over and above risk factor awareness education and lifestyle advice alone [3]. There is also increasing interest in the potential benefits of incorporating risk stratification into cancer screening programmes to enable the screening frequency, modality, and/or eligible age range to be adjusted to potentially optimise the benefit-harm ratio [4].

However, the general population does not easily understand the concept of risk [5,6], with lay perceptions of risk often being resistant to change and differing substantially from those of experts [7]. These discrepancies are potentially consequential. Risk perception, particularly when assessed using high quality measures, has been shown to predict behaviour [8], and cancer risk perception specifically is associated with health-related quality of life, depression, anxiety and cancer worry [[9], [10], [11]]. Understanding the impact of providing personalised cancer risk information on perceptions of risk and psychological responses is, therefore, important.

Previous reviews have shown that provision of cancer-based risk information in genetic counselling centres can increase accuracy of risk perception while leading to either no change in psychological outcomes or psychological benefits [[12], [13], [14]]. Individuals attending genetic counselling centres, however, are typically referred by healthcare professionals due to a family or personal history of cancer. These individuals are, therefore, already aware that they are potentially at high risk and their responses to risk information may differ from those at population level risk. To inform future population-based communication of cancer risk, we aimed to synthesise the effects of interventions incorporating non-genetic personalised cancer risk information on accuracy of risk perception and psychological responses in individuals not already identified as at high risk on the basis of a personal or family history of cancer or following referral to specialist cancer risk services.

## 2 Methods

We performed a systematic literature review following an *a priori* established study protocol (available on request). Reporting followed the PRISMA statement [15].

### 2.1 Search strategy

We used the same search strategy as for a previous review of the effect of interventions incorporating personalised cancer risk information that focused on intentions and behaviour [16]. This included an electronic literature search of Medline, EMBASE, CINAHL and PsycINFO from 1^st^ January 2000 until 1^st^ July 2017 with no language limits, using a combination of subject headings and free text incorporating ‘cancer’, ‘risk/risk factor/risk assessment’ and ‘prediction/model/score/tool’ (see Appendix File A.1 for the complete search strategies). We manually screened the reference lists of all included papers to identify additional papers. As the outcomes of interest for this review are not collected routinely within healthcare and both CINAHL and PsycINFO include citations to books, reports, dissertations and theses, we did not specifically search for additional grey literature.

### 2.2 Study selection

We included studies if they met the following criteria: 1) were published as a primary research paper in a peer-reviewed journal; 2) included adults with no previous history of cancer; 3) included provision to individuals of a personal estimate of future cancer risk based on two or more non-genetic variables, either alone or as part of a larger intervention; and 4) included data on either accuracy of risk recall or risk perception at the level of the individual or psychological measures (including cancer worry, anxiety, depression, affect and quality of life). As in our previous review [16], in order to focus on the provision of personalised cancer risk information to the general population, we excluded studies that had recruited participants on the basis of a personal or family history of cancer or following referral to specialist cancer risk services. We also excluded vignette studies, qualitative studies, conference abstracts, editorials, commentaries and letters.

Two reviewers (JUS and BS) each screened half of the titles and abstracts to exclude papers that were clearly not relevant. A third reviewer (SG) independently assessed a random selection of 5% of the papers screened by each of the first reviewers. The full text was examined by two reviewers (MB and MF) independently if a definite decision to exclude could not be made based on title and abstract alone. A third reviewer (JUS) then assessed all those for which it was unclear at full text level whether or not the inclusion criteria were met.

### 2.3 Data extraction

At least two researchers (JUS/BS/MB/MF) independently extracted data from studies included in the review directly into data tables. This included data on: (1) study characteristics (cancer type, study design, study setting, duration of follow-up); (2) selection of participants (inclusion criteria, method of recruitment/randomisation); (3) participant characteristics (age, level of cancer risk, sample size); (4) the intervention (risk tool used, method and format of risk communication, additional information or follow-up provided), and (4) measured outcome(s). Reviewers were not blinded to publication details. If numerical data were not included in the published articles, we wrote to the authors requesting additional information.

### 2.4 Quality assessment

Quality assessment was performed by two reviewers (MB and MF) using a checklist based on the Critical Appraisal Skills Programme (CASP) guidelines [17]. This includes eight questions concerning whether the study addressed a clearly focused issue, the method of recruitment and randomisation, whether blinding was used, the measurement of the exposure and outcome, the comparability of the study groups and the follow-up. Each study was then classified as high, medium or low quality. We did not exclude any studies based on quality alone.

### 2.5 Data synthesis and statistical analysis

As data on psychological outcomes (worry, anxiety, fear, depression and quality of life) used different measurement scales and variably reported change from baseline to follow-up and mean values at follow-up, it was only possible to pool results for accuracy of risk perception. For the comparison between risk information and no information we used random effects meta-analysis [18] and the ‘metan’ package in Stata and present intervention effects as relative risk (RR) rather than odds ratios (OR) to avoid overestimating the risk [19]. If there were zero participants in any group, we added 0.5 to each of the cells of the 2 × 2 table in both the control and intervention group [20]. For the study by Timmermans et al. [21] in which data were reported for accuracy in the same participants for colon cancer and lung cancer separately, we included only the results for colon cancer in the meta-analysis to avoid including the same participants twice in the same analysis. The results were similar when lung cancer was included instead (data not shown). To pool the percentage who were able to recall the risk information provided to them accurately and those whose risk perception accurately matched the risk estimate that they had been provided we also used the ‘metan’ package in Stata with a random effects model. In both cases we quantified the heterogeneity between studies using the I^2^ statistic. All analyses were conducted using statistical software package Stata/SE version 14.

## 3 Results

**Fig. 1:**
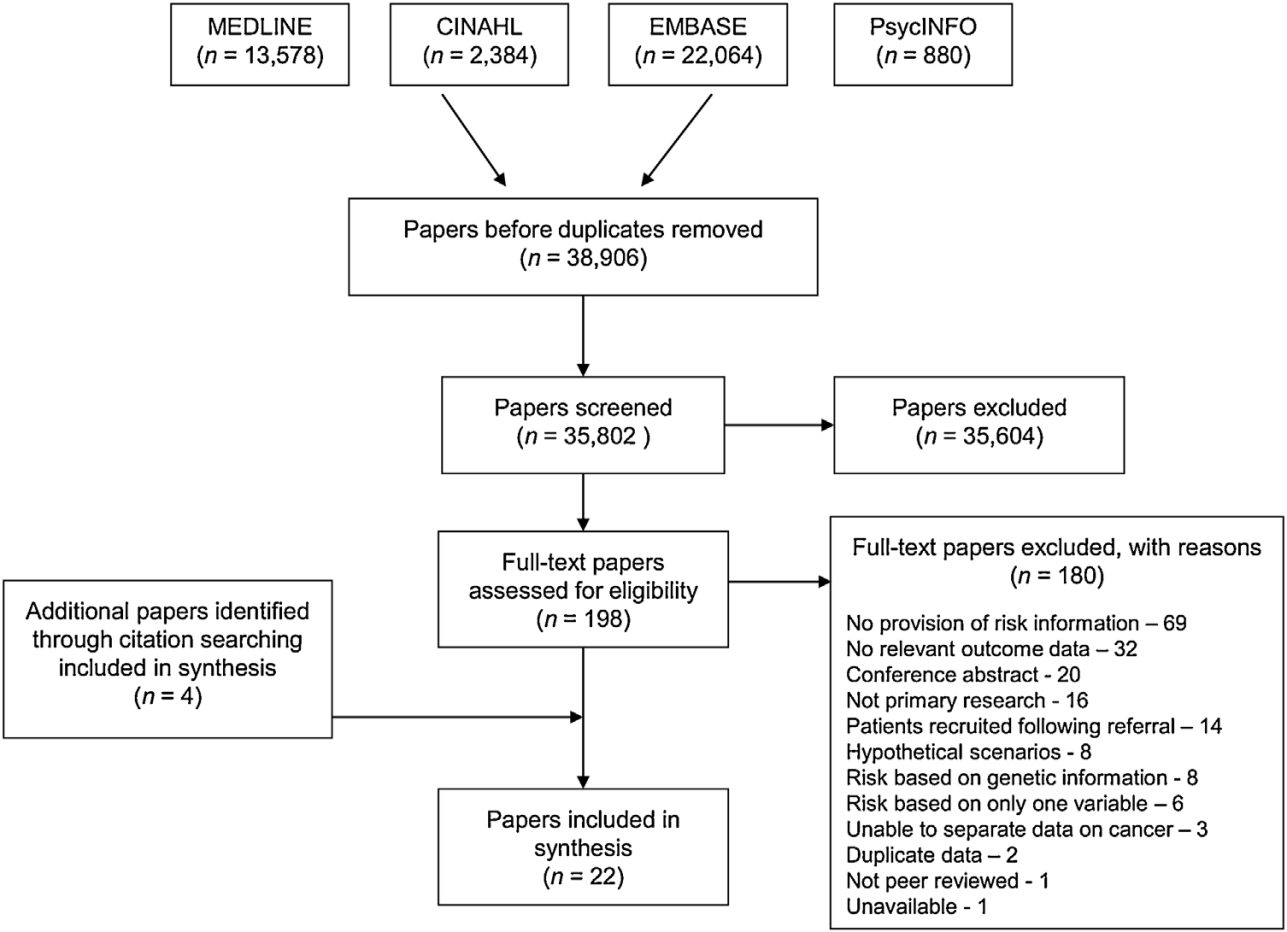
PRISMA Flow diagram.

As reported previously [16], we identified 35,802 unique papers from the electronic search. Of these, 35,604 were excluded at title and abstract level. After screening by the first reviewer (JUS/BS), no additional papers met the inclusion criteria in the random 5% screened by the second reviewer (SG). A further 180 were excluded after full-text assessment against the inclusion and exclusion criteria specific for this review question. The most common reasons for exclusion at this stage were that the papers did not include provision of a personal risk estimate (*n* = 69), did not include any data on predefined outcomes (*n* = 32), were conference abstracts (*n* = 20), or were not primary research (*n* = 16) (Fig. 1). We identified four additional papers through citation searching, leaving us with 22 papers describing 23 studies in the analysis.

Table 1 summarises the design, setting and key outcomes of the 23 included studies and Table 2 provides additional details about the tools used to estimate the personalised risk and the format in which the risk information was provided. The majority (*n* = 15) focused on provision of breast cancer risk derived from the Gail model [22], four provided risk information about colorectal cancer, one lung cancer, one cervical cancer, one colorectal and lung cancer, and one colorectal, breast and ovarian cancer. All but two studies [23,24] were conducted in the USA. Twelve were assessed as high or medium/high quality, seven as medium quality and four as medium/low based on the CASP guidelines (Appendix File A.2) [[25], [26], [27], [28], [29], [30]].

**Table 1:** Details of the setting and key outcomes of the included studies.

Author, year	Cancer site(s)	Design	Follow-up	Setting and participants	Risk level / co-morbidities	Outcome(s)	Quality*
Bowen 2006	Breast	RCT	6 and 24 months	150 sexual minority women recruited via public advertisements	Mean Gail lifetime risk 12%	Quality of life and cancer worry	H
Bowen 2010	Breast	RCT	12 months	1,366 women recruited via telephone with no previous diagnosis of breast cancer	Mean Gail lifetime risk 12%	Quality of life	H
Davis, 2004	Breast	RCT	1 month	392 women with no history cancer calling the Cancer Information Service	27% 2-6% lifetime risk; 32% 6-9% lifetime risk; 41% 9-46% lifetime risk	Accuracy of risk perception and cancer worry	M
Dillard, 2006a	Breast	RCT	0, 2 weeks	Convenience sample of 72 female undergraduates with no first degree relatives with breast cancer	Not given	Accuracy of risk perception, cancer worry	L-M
Dillard, 2006b	Breast	RCT	0, 2 weeks	Convenience sample of 62 female undergraduates with no first degree relatives with breast cancer	Not given	Accuracy of risk perception, positive and negative affect and cancer worry	L-M
Emmons, 2004	Colorectal	RCT	0	353 patients with no history of cancer scheduled for routine or non-urgent health care visits to two primary care practices	Mean 20 year risk 9.96 per 1,000	Accuracy of risk perception and cancer worry	M-H
Helmes, 2006	Breast	RCT	3 months	Random sample of 340 members of state healthcare system with no history of breast/ovarian cancer or testing for cancer risk	Mean 9.5% (3.2) lifetime risk	Accuracy of risk perception and cancer worry	M-H
Holloway, 2003	Cervical	RCT	0, 4 years	1890 women attending routine cervical smear test at one of 29 GP practices	78-80% very low risk; 20-22% low risk	Accuracy of risk perception, 21 short-term outcome measures relating to knowledge and psychosocial wellbeing.	H
Lipkus 2006	Colorectal	RCT	0	160 members of general public with no history of CRC or screening for CRC recruited through newspaper advertisements	Not given	Accuracy of risk perception and cancer worry	M
Lipkus, 2001and klein	Breast	2 × 2 design	0, 6-8 months	169 members of general public recruited through newspaper advertisements	Mean lifetime risk 7.78% (SD 1.13)	Cancer worry	M-H
Lipkus, 2001b	Breast	RCT	0	121 members of general public recruited through newspaper advertisements	Mean 10 year risk 2.65% (SD 1.13)	Negative affect related to getting breast cancer and accuracy of risk recall	M
Lipkus, 2005	Breast	RCT	0	301 members of general public recruited through newspaper advertisements	Mean lifetime risk 8.5% (range 1.2 to 30.5)	Accuracy of risk perception and cancer worry	M
Livaudais-Toman, 2015	Breast	RCT	1 week	1235 women with scheduled appointments at an academic medical centre or hospital with no history of breast cancer	25% high risk	Accuracy of risk perception and cancer worry	H
McCaul, 2003	Breast	2 × 2 design	0, 1-2 weeks	59 female undergraduates with no first-degree relatives with breast cancer at one university	Mean lifetime risk 11.5%	Accuracy of risk perception, cancer worry	M
Quillin, 2004	Breast	RCT	1 month	299 women with no history of breast cancer attending outpatient mammography clinic	Mean lifetime risk 11.1% (SD 5.14)	Accuracy of risk perception	M
Rimer, 2002	Breast	RCT	1 and 2 years	752 women aged 40-44 and 50-54 enrolled in a personal care plan	Mean 10 year risk 2.7%	Accuracy of risk perception	M-H
Seitz, 2016	Breast	RCT	0	2918 women aged 35–49 with no history of breast cancer or BRCA1 or BRCA2 mutation recruited through a survey company	42 % had a 10 year risk of <1.5% and 58% had a risk of >1.5%(mean 2.53 SD 0.04)	Accuracy of risk perception	M-H
Sherratt, 2016	Lung	RCT	6 month follow up	Participants were aged 18 to 60 years, and participants were excluded from the project if they had previously been diagnosed with lung cancer. 297 current and 216 recent former smokers aged 18– 60 years without a history of lung cancer and attending smoking cessation services	Not given	Cancer worry	H
Timmermans 2012	Colon, lung	RCT	0	612 members of general public with no history of cancer	4.6% reported a history of cancer	Accuracy of risk perception and cancer worry	M
Trevena 2008	Colorectal	RCT	1 month	314 patients recruited from 6 primary care practices without a history of colorectal cancer	Not given	Anxiety related to cancer	M-H
Van Erkelen, 2017	Breast,	RCT	0, 2 weeks	287 women aged 50-74 with no previous history of BC or diagnosis of increased BC risk, recruited from routine population-based screening	95% population risk, 1% moderately high risk, 4% high risk	Accuracy of risk perception, state and trait-anxiety and distress score related to cancer	L-M
Wang, 2012	Colon, breast, ovarian	RCT	6 months	3786 patients from primary care clinics with no history of colon, breast or ovarian cancer invited by mail following record review	82% moderate or strong risk for ≥1 of the 6 conditions	Accuracy of risk perception	H
Weinstein, 2004	Colon	2 × 2 design	0	353 patients with no history of cancer with scheduled routine or non-urgent health care visits at two primary care practices	Below-average	Accuracy of risk perception and accuracy of risk recall.	L-M

RCT – randomised controlled trial; CRC – colorectal cancer; CT computerised tomography.

* L – low, M – medium, H – high.

**Table 2:** Details of the risk-based interventions in each of the included studies.

Author, year	Risk tool	Intervention group(s)	Comparison (where applicable)	Format of risk
Bowen 2006	Gail model (5 year, 10 year and at age 79)	Four weekly 2 -h sessions led by a health counsellor focusing on risk assessment and education, screening, stress management and social support	Delayed intervention	No details given
Bowen 2010	Gail model (lifetime)	Information sheets with general information on breast cancer risk and personalised risk information plus telephone counselling and offer for more intensive group or genetic counselling	Delayed intervention	Bar graph of absolute lifetime risk along with age-appropriate estimates for the “average risk” woman
Davis, 2004	BRCA tool (updated version of Gail model) (lifetime)	10 min brief intervention designed to increase accuracy of perceived risk including results of risk assessment and screening recommendations tailored to participant's stage of adoption of mammography and follow up written information	No intervention	Verbal over the telephone. No additional details given.
Dillard, 2006a	Gail model (5 year and lifetime)	Risk feedback sheet following completion of risk assessment questions plus kindness questionnaire or study calendar +/- additional questions about risk factors	No intervention	Absolute risk estimate as % and comparative estimate ranging from 'much lower' to 'much higher' along with a visual scale with risk estimate represented by a mark on the scale
Dillard, 2006b	Gail model (5 year and lifetime)	Risk feedback sheet including information on two other women and their risk factors as downward social comparison condition	Risk feedback sheet	Absolute risk estimate as % and comparative estimate ranging from 'much lower' to 'much higher' along with a visual scale with risk estimate represented by a mark on the scale +/- downward social comparison condition
Emmons, 2004	Harvard cancer risk model (20 year)	1) Absolute risk with active impact; 2) Absolute risk without active impact; 3) Absolute and relative risk with active impact; 4) Absolute and relative risk without active impact	Passive risk communication but no absolute or relative risk estimates	Absolute risk over 20 years +/- relative risk plus absolute risk +/- option to manipulate their risk factor profiles to see impact of changing risk factors on a visual scale using an interactive computer-based tool
Helmes, 2006	Gail model (lifetime)	Face-to-face or telephone intervention consisting of 8 items: 1) a personal risk sheet ; 2) a personal computer-drawn pedigree; 3) a 23 page participant booklet; 4) Breast self-examination brochure; 5) Pap smear and mammography brochure; 6) BSE shower card; 7) pictures of chromosomes and gene mutations; 8) a list of community resources for breast cancer	No intervention	Bar charts of absolute % risk with numerical % alongside for the individual, an average-risk woman, and a high-risk woman
Holloway, 2003	Wilkinson score	Brief 10 minute counselling session integrated with smear test appointment including relative and absolute risks and then negotiation of appropriate screening intervals	Normal care	Comparative and absolute risk in pictures and numbers
Lipkus 2006	Not given	Written information about CRC, CRC screening methods and CRC risk factors plus either 1) tailored CRC risk factor information or 2) tailored CRC risk factor information plus information on whether their total number of CRC risk factors was greater or not than average	Written information about CRC, CRC screening methods, and CRC risk factors	Narrative comparative risk
Lipkus, 2001a	Gail model (lifetime)	1-2 page handout describing the Gail Model plus either 1) a point estimate of their risk; 2) a risk range derived from the 95% confidence intervals; 3) a point estimate of their risk plus a risk range derived from the 95% confidence intervals	No information	As a percentage in a pie chart
Lipkus, 2001b	Gail model (10 year)	1 page handout describing the Gail model plus absolute risk alone	As for intervention group plus how their risk compared to a woman of their age and race at the lowest level of risk	Absolute risk +/- risk of a woman at the lowest level of risk as percentages in a pie chart
Lipkus, 2005	Gail model (lifetime)	In three groups, women obtained information about their absolute risk only, in one of three formats. Three additional groups received their absolute risk in one of the three formats along with information about the risk of another woman the same age and race as the participant with no other risk factors	No information	Numerical percentages either 1) “point estimate condition’’ - single best point estimate of their risk as a percentage; 2) “range condition’’ - upper and lower bounds of risk as percentages; 3) "point estimate and range’’
Livaudais-Toman, 2015	Referral Screening Tool; Gail Model; and Breast Cancer Surveillance Consortium model (5 year)	Individually-tailored print-outs for patients and their physicians (one page in length) including specific risk reduction recommendations.	No information	Absolute risk as a percentage and comparative risk (higher/lower)
McCaul, 2003	Gail model (5 year and lifetime)	Printed feedback on two sheets including either absolute risk information, relative risk information, or both	No information	Absolute risk as a percentage and mark on two scales ranging from 0% to 100%. Comparative risk as a label (e.g., ‘Same’) and a mark on a scale ranging from ‘Much lower’ to ‘Much higher,’ with seven labels including a centre label of ‘About the Same’
Quillin, 2004	Gail model (5 year and lifetime)	Risk assessment with genetic counsellor then one-page summary including breast health messages that were appropriate for their calculated risk, including recommendations for screening, available genetic counselling, and contact information for psychosocial support	No information	Percentage risk alongside qualitative interpretation ("low", "moderate", high") and whether it is higher/lower than the average women's risk
Rimer 2002	Gail model (10 year and lifetime)	Tailored print booklet and brief tailored newspaper plus personalized risk	Usual care (postcard reminder)	Absolute risk as a percentage
Seitz et al 2016	NCI BRCAT – based on the Gail model (10 year)	Online risk plus basic information about mammography and national recommendations plus either (1) statements about women making choices, (2) untailored examples of women making choices or (3) examples of similar women making choices	No information or the same basic information as intervention group	All received Individualized 10-year and lifetime estimates of their objective risk for developing BC and the risk of an average-risk age-matched woman, all presented as both numeric frequencies and icon arrays.
Sherrat et al 2016	Liverpool lung project risk model (5 year at age 70)	Personalised risk plus booklet stating the association between smoking and lung cancer and highlighting that quitting smoking was the best thing to do	As for intervention but without personalised risk assessment	Verbal and written absolute risk if continue to smoke and if stop smoking alongside icon arrays
Timmermans 2012	Shortened KWF Kanker Risico Test (5 year)	Participants were randomized to one of 12 experimental groups who received a combination of: 1) Average population risk (no quantitative risk information provided/only the number/number + graphic illustration); 2) the calculated personal risk (no quantitative information /numbers); and 3) the relative risk reduction after changing lifestyle (or no quantification of risk reduction)	Standard version of the KWF-KRT	12 different formats including numbers, graphical illustrations (emoticons and bar charts) of average population risk, personal risk and relative risk reduction
Trevena 2008	No details given	20 page booklet including personalized risk, absolute reduction in colorectal cancer mortality with screening over the next 10 years, probability of test outcomes from screening and information about how to get screened.	3 page booklet with information and recommendations about screening	Words and 1000-face diagrams
Van Erkelen, 2017	Dutch BC guidelines	Patients given information that assigns them to 1 of 3 risk groups: high risk in need of genetic counselling, moderate risk in need of earlier screening or population risk.	Statistical analysis used comparison between assigned risk groups	Assignation to 1 of 3 risk groups: high, moderate or population.
Wang, 2012	Family Healthware tool	Written personalized prevention messages delivered via mail, e-mail, or in person tailored to familial risk for each of the six conditions alongside a family tree and information about the characteristics in one’s family history that put the person at increased risk (if applicable)	Standard print messages about screening and lifestyle choices via mail, e-mail, or in-person	Qualitative risk - weak, moderate or strong familial risk
Weinstein, 2004	Harvard cancer risk model (20 year)	Absolute or relative risk electronically +/- the opportunity to manipulate the risk along with details of the risk factors that comprised their risk and recommendations for what they should change to reduce their risk	Feedback on which of their behaviours and non-modifiable attributes lowered and which increased their risk and advice on steps they could take to lower their risk	Absolute risk - numerical estimate in units of cases per thousand people like them alongside an oval window with the risk marked on a horizontal hairline. Comparative risk was expressed in terms of one of seven categories: “very much below average’’, “much below average,’’ “below average,’’ “average’’, “above average,’’ “much above average,’’ and “very much above average’’ alongside an oval window with the risk marked on a horizontal hairline

CRC – colorectal cancer.

### 3.1 Recall of risk information

**Fig. 2:**
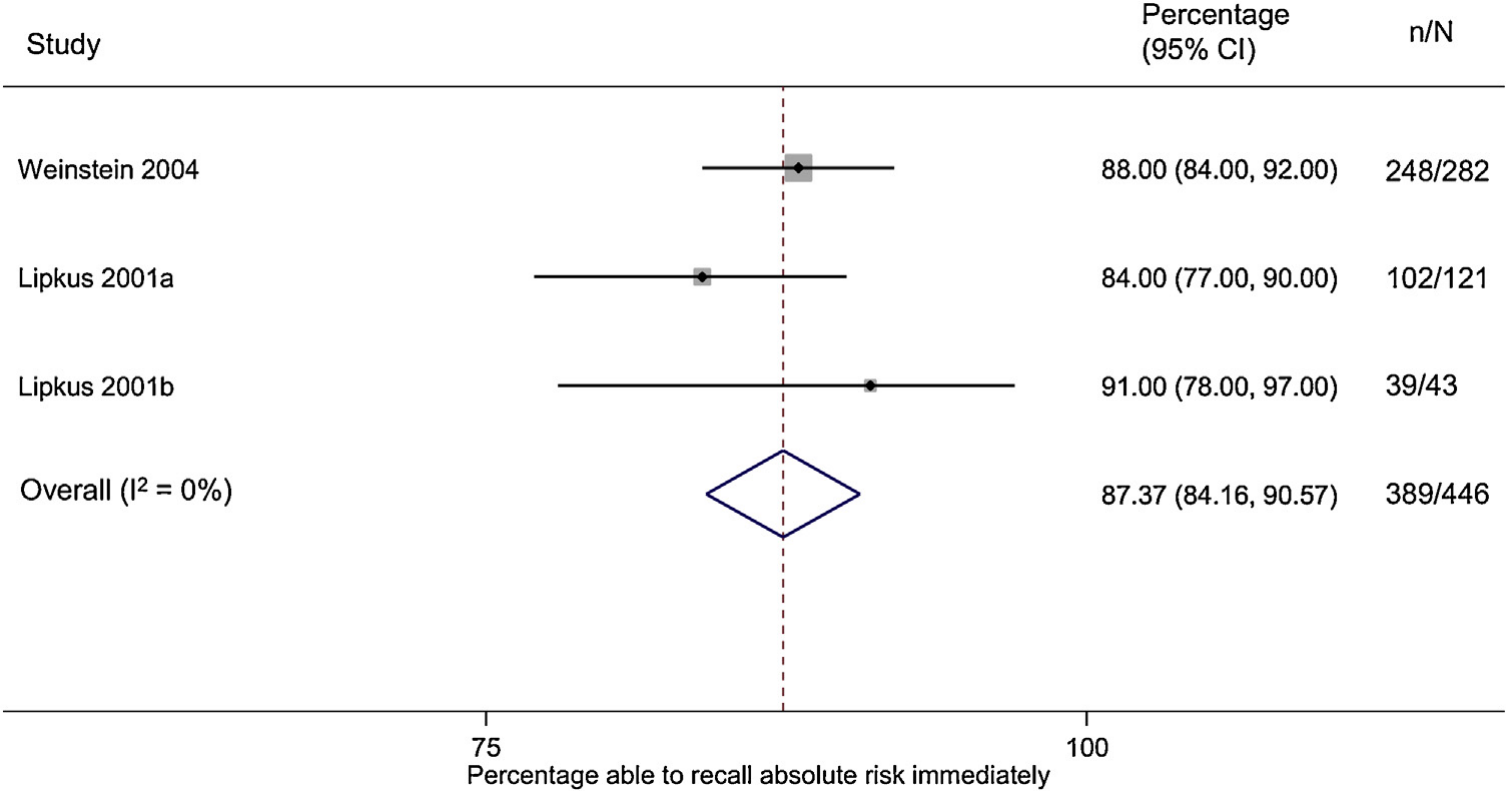
Forest plot showing the percentage of participants able to accurately recall the absolute risk estimate immediately after receiving risk information.

Three studies reported recall of absolute risk [[31], [32], [33]]. Immediately after being provided with risk information, 87% (95% CI 84%–91%, I^2^ = 0%) of those given absolute risk information were able to recall their numerical absolute risk estimate accurately (defined as exact agreement) (Fig. 2), with no difference between those presented risk of breast cancer as either a point estimate on a 0–100% scale, as a range, or as a point estimate plus a range [33]. Comparative risk, where individuals were provided with estimates of their risk in comparison with others, was reported in only one study where 64% were accurate [31].

Two of these studies additionally compared recall of risk information and risk perception. In the study by Lipkus et al. only 17% (*n* = 19/102) of those who were able to recall their risk estimate perceived that to be their risk within 0.5%, with 71% (*n* = 72/102) believing their risk to be higher and 12% (*n* = 12/102) their risk to be lower [32]. Similarly, in Weinstein et al., those who had received absolute risk information gave the same answer for their own beliefs as their recollection of what they had been told only 45% of the time, giving a higher value for their own beliefs 47% of the time and a lower value 8% of the time [31]. Corresponding percentages for comparative information were 39%, 46% and 15% respectively. A further study did not compare recall with perceived risk but instead asked women at a follow-up telephone interview how they would compare their actual risk with the estimate provided in the study. 53% thought that their actual risk was ‘just the same,’ while 38% thought that their risk was greater than what they had been told [34].

### 3.2 Accuracy of risk perception

Thirteen studies reported data on accuracy of risk perception. Eight of these reported accuracy as the agreement between the perceived risk estimates participants gave and the estimated personalised risks they had been presented with. The other five studies reported accuracy indirectly, either as the extent of overestimation or the change in risk perception in groups known to all either over-estimate or under-estimate their risk at baseline.

**Fig. 3:**
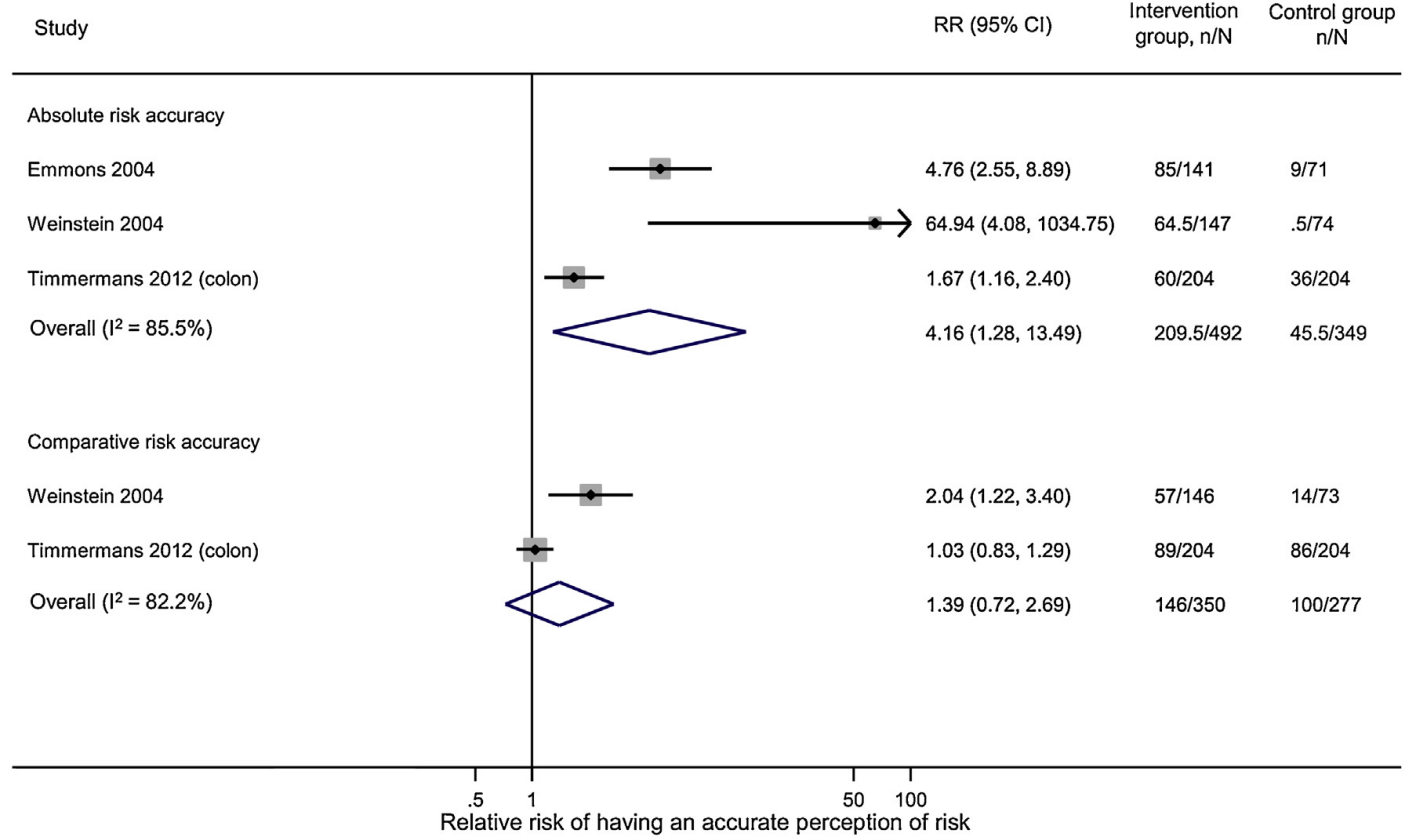
Forest plot showing the relative risk of having an accurate perception of absolute or comparative risk immediately after receiving it compared to controls who did not receive risk information.

Definitions of what constituted “accurate” and the time interval between provision of risk information and follow-up varied widely between studies (Table 3). This made pooling many of the results inappropriate. It was possible, however, to pool data from three studies that measured accuracy of absolute or comparative risk perception immediately after provision of risk information about colon cancer compared with no information [21,31,35]. Those who received both absolute and comparative risk estimates were more likely to have accurate absolute risk perceptions immediately post risk information (pooled RR 2.59 (1.40 to 4.81) I^2^ = 81.2%) (Fig. 3), with no difference between those provided with absolute risk alone or absolute plus comparative risk (data not shown). There was no significant effect on comparative risk accuracy (pooled RR 1.11 (0.74 to 1.66) I^2^ = 82.9%) (Fig. 3).

**Table 3:** Summary of findings for accuracy of risk perception across the included studies.

Author, year	Definition of accuracy	Time	Main finding	Effect
**Agreement between risk perception and risk estimates**				
Absolute risk				
Weinstein 2004	Exactly the same number	0	Those who received both absolute and comparative risk estimates were more likely to have accurate absolute risk perceptions immediately post risk information (pooled RR 2.59 (1.40 to 4.81) I2 = 81.2%), with no difference between those provided with absolute risk alone or absolute plus comparative risk	↑
Emmons 2004	Within 0.5%	0		
Timmermans 2012	Within 2%	0		
Lipkus 2005	Within 5%	0	No difference between a control group and women who received either absolute or comparative risk information, with no effect of age, race or education	↔
Rimer 2002	Within 10%	1 and 2 years	Women were more likely to be accurate at follow-up if they had been accurate at baseline (OR = 7.0 (4.9-10.0), p < 0.001); received tailored print materials including personalised breast cancer risk estimates plus telephone counselling vs control (OR = 2.1 (1.4-3.3), p < 0.001. There was no increase in accuracy among those who just received printed information compared with control (OR = 1.0 (0.6-1.6), p = 0.96). No differences were seen with race/ethnicity or educational level.	↑
Comparative risk				
Livaudais-Toman 2015	Two groups - below average or average and above average	1 week	No difference between a control group and one that received comparative risk information ([OR] = 0.98; [CI] = 0.72–1.33), % accurate at follow-up 70% control and 66% intervention, p = 0.11)	↔
Wang 2012	Two groups - below average or average and above average	1 week	Among those who underestimated risk at baseline, a greater percentage of those who received their personalised risk increased their risk perceptions at the 6 month follow up compared to individuals in the control arm for colon cancer (17% vs 10%,OR 1.89 (0.99 to 3.59), p = 0.05), but not for breast cancer or ovarian cancer (OR 1.48 (0.61 to 3.58) and OR (0.10 to 2.59) respectively)	↑
Timmermans 2012	Three groups - below average, average and above average	0	No significant effect (pooled RR 1.11 (0.74 to 1.66) I2 = 82.9%)	↔
Lipkus 2005	Bias in comparative risk*	0		
Quillin 2004	Two groups - below average or average and above average	1 month	Significant change from baseline to follow-up from 78.7% (n = 107) to 85.3% (n = 99), p < 0.01	↑
Quillin 2004	Three groups – ‘usual’ risk for an estimated lifetime risk <15%, ‘moderate’ risk for 15-30% and ‘strong’ risk for >30%	1 month	No significant change from baseline to follow-up (% accurate 65.2% (n = 88) pre-intervention and 68.1% (n = 77) post intervention, p = 0.46)	↔
**Overestimation as a measure of accuracy**				
Davis 2004	Percentage overestimating their risk	1 month	No difference (-2.7% in the control group (n = 184) compared with -5.8% in the intervention group who received a 10-minute educational intervention over the telephone (n = 183), p = 0.20). However, among women with a first-degree family history of breast cancer, those in the intervention group significantly reduced their risk overestimate compared to those in the control group (-12.5 vs. 2.8, p = 0.006).	↔↑
Seitz 2016	The degree to which participants overestimated their risk	0	Consistent improvement across six intervention groups when risk was measured as a percentage but not when risk was measured as a frequency out of 1000. For women with an estimated risk <1.5%, this effect was moderated by numeracy, with women with high numeracy having greater increases in accuracy than women with low numeracy. No significant moderation effects were seen for women with an estimated risk ≥1.5%.	↑
**Indirect assessment of accuracy in populations who all overestimated risk at baseline**				
Dillard 2006a	---	0	The mean estimate of absolute risk among 72 undergraduate women decreased from 56.4% to 28.4% two weeks after absolute and comparative risk information. These, however, remained significantly higher than the estimated risk (mean 11.2% difference) p < 0.01. No significant differences were seen among those who were asked to provide a pre-intervention risk estimate, those who were led to believe that all the factors they considered possibly responsible for their own breast cancer risk were used to compute their risk, or those who completed a self-affirmation task.	↑
Dillard 2006b	---	2 weeks	Participants provided with their risk alone and those provided with their risk plus social comparison conditions reduced their risk estimates from pre-test to post-test, and maintained their new estimates at the 2-week follow-up (pre-test mean 48.1% (SD 18) and 44.8% (SD 15.8) and post-test means 26.8% (SD 20.5) and 16.9% (SD 11.2) for those in a risk only and risk plus social comparison groups respectively). Their estimates remained higher than the estimated risks they had been given (mean 16.9% vs 10.9%, p < 0.001).	↑
McCaul 2003	---	1 week	Women who received absolute risk reported both lower absolute risk perceptions (mean 34.9% compared with mean 52.1%, p < 0.01) and lower comparative risk perceptions (mean 4.10 compared with mean 4.43, p = 0.05) immediately and at one week follow-up than women who did not. The effect for comparative risk information was not quite significant (p = 0.07) but women who received comparative risk estimates did report lower risk (mean 4.11) than those who did not (mean 4.43)	↑

* Computed by first subtracting the participants’ personalised risk estimate from the risk estimate of the average same-aged woman with no risk factors, then subtracting participants’ estimates of their own and the average woman’s absolute numerical risk, and then comparing the two differences and categorising participants as accurate if the differences were within 5%.

**Fig. 4:**
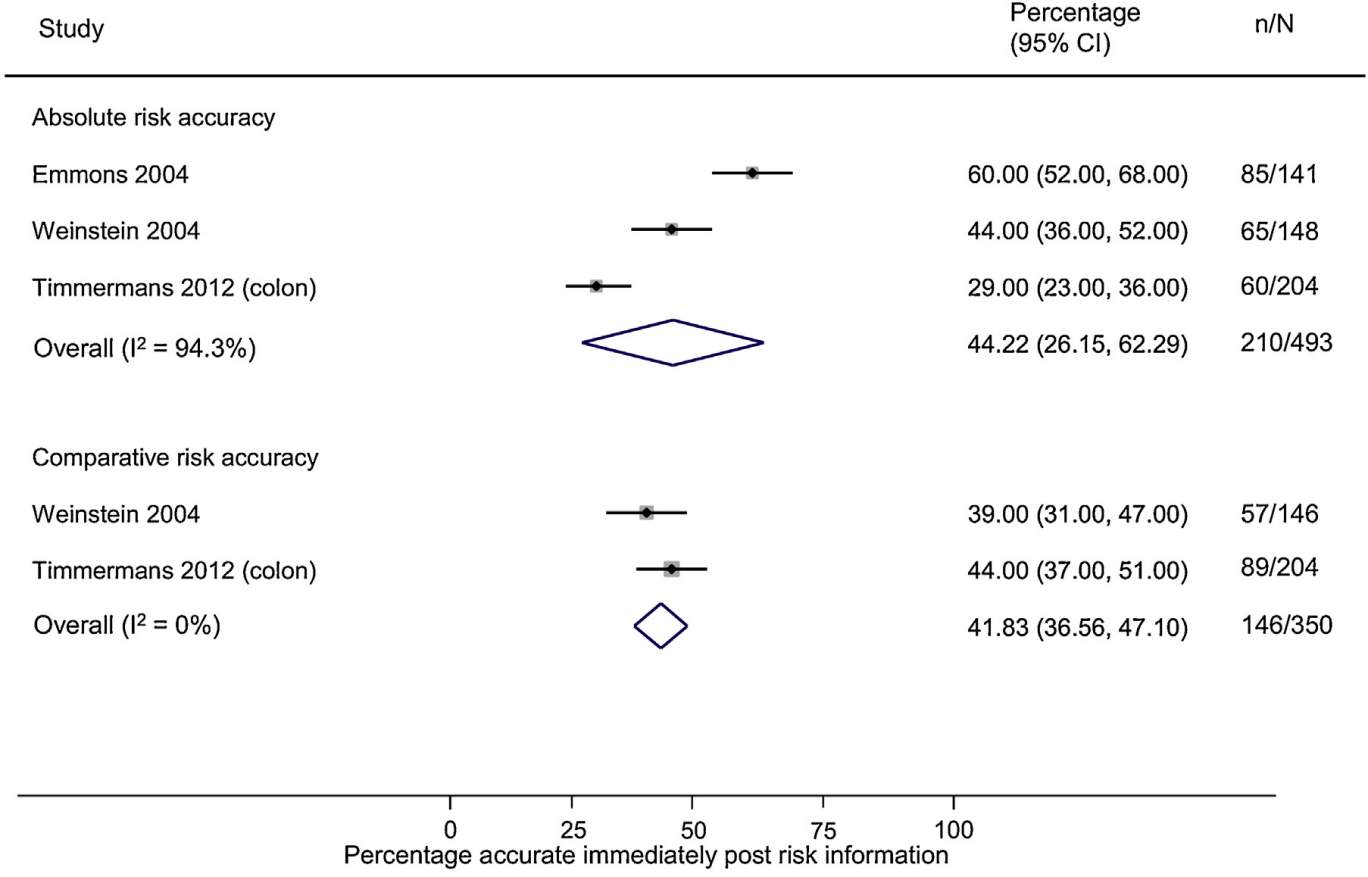
Forest plot showing the percentage of participants who had an accurate perception of their personal absolute or comparative risk after receiving it.

Despite these improvements in accuracy compared to control groups, even immediately after risk information, up to half of all participants remained inaccurate (pooled percentage for absolute risk accuracy 44% (31%–56%, I^2^ = 91.5) and for comparative risk 40% (95% CI 36%–44%) (Fig. 4).

The findings from these and the other studies that could not be pooled are summarised in Table 3. Overall, eight showed improvements in accuracy, two no effect and three mixed results. One study directly compared the effect of alternative formats on risk accuracy. In that study, Emmons et al. showed that those who were randomised to have the opportunity to see how adopting or changing any of the risk factors would impact on their total risk profile had greater improvement in accuracy immediately post information for both comparative and absolute risk accuracy compared to those who did not [35]. A further study assessed the role of numeracy and found that among women with an estimated risk <1.5%, the degree to which participants overestimated their risk was moderated by numeracy, with women with high numeracy having greater increases in accuracy than women with low numeracy [36]. No significant moderation effects were seen for women with an estimated risk ≥1.5%.

Having the opportunity to see how changing any of the risk factors would influence their risk, as well as inclusion of social comparison information [37], appeared to be associated with greater improvements in accuracy of perceived risk. By comparison no differences were seen for providing pre-intervention risk estimates, self-affirmation, providing data so that individuals believed that all factors they considered possibly responsible for their own risk were used to compute their risk [37], or with race or education level [38,39].

### 3.3 Psychological responses

#### 3.3.1 Cancer specific worry, anxiety or fear

Thirteen randomised controlled trials (RCTs) reported cancer specific worry, anxiety or fear. As the studies used different scales and variably reported change from baseline to follow-up and mean values at follow-up, it was not possible to pool the studies. Instead, the findings are summarised in Table 4. Ten reported no significant change and three a reduction.

**Table 4:** Summary of findings for worry across the included studies.

Author, year	Measure of worry	Main finding	Effect
**Change from baseline to post-intervention**			
Bowen 2006	Lerman four item cancer worry scale	Significant decrease in worry among the group that received genetic counselling from 5.9 (SD 2.0) to 5.2 (SD 1.5) at six months and 5.2 (SD 1.6) at two years (both p < 0.001)	↓
Helmes 2006	Lerman four item cancer worry scale	Significant decrease (p < 0.001) in worry among women who received both absolute and comparative risk information either in-person or telephone counselling when compared to a control group who received no information (the control arm decreased from 5.48 to 5.10, the in-person arm from 5.61 to 4.71, and the telephone arm from 5.50 to 4.68)	↓
Davis 2004	12-point scale adapted from the Lerman scale	No difference in the change in breast cancer worry from pre- to post-test between women who received absolute risk information over the telephone and a control group who received no information ((-0.17 vs -0.24, p = 0.65)	↔
Emmons 2004	5-point scale from ‘*much more worried*’ to ‘*much less worried*’	No increase in worry across any of four intervention groups that received either absolute plus comparative risk or absolute risk alone with or without the option to manipulate the risk factors and see the impact of that on their risk. At follow-up 33% (n = 116) reported being less worried about getting colorectal cancer and 17% (n = 61), all of whom had perceived comparative risks of below average or lower at baseline, reported being more worried	↔
Livaudais-Toman 2015	Single question - ‘*How concerned are you about getting breast cancer?*’	No change in the proportion ‘*very concerned*’ from baseline to follow up among controls (22.3% vs 22.0%, *n=*655) and a slight but non-significant decrease among women who received absolute and comparative risk information (27.1% vs 24.2%) (OR = 0.94, 95% CI 0.69–1.28)	↔
**Differences between groups post intervention**			
McCaul 2003	Single question - ‘*How worried are you about developing breast cancer?*’	No significant difference in post-intervention worry adjusted for baseline worry immediately and one to two weeks after being provided with absolute risk information. A significant reduction in worry was seen among those provide with comparative risk information (p < 0.01)	↔
Sherratt 2016	Single question - ‘*How often are you worried about lung cancer?*’	No change in the proportion who were worried ‘*Often or all the time*’ compared to ‘*Rarely or never*’ at six months follow-up among those provided with absolute risk information compared with a control group both amongst current smokers (*p* = 0.869) and recent ex-smokers (OR 2.18 95% CI 0.79-6.00, *p* = 0.274)	↔
Dillard 2006	Single question - ‘*How worried are you about developing breast cancer?*’	No significant differences were found between women who were asked to provide a pre-intervention risk estimate, those who were led to believe that all the factors they considered possibly responsible for their own breast cancer risk were used to compute their risk, or those who completed a self-affirmation task, or between those provided with their risk alone and those provided with their risk plus social comparison	↔
Timmermans 2012	Percentage who agreed or disagreed with the statement ‘*I am more worried now about my risk of cancer than before I did my cancer risk test*’	After receiving a combination of information on average population risk, personal risk and the relative risk reduction after changing lifestyle, 55.4% of participants disagreed with the statement for colon cancer and 61.4% for lung cancer and 12.1% and 11.18% agreed for colon cancer and lung cancer respectively, indicating that worry had stayed the same or reduced in most individuals	↔
Lipkus 2005	Combined responses to three questions about how worried, fearful and anxious they were about developing breast cancer	No difference between participants provided with either no risk information or absolute or absolute plus comparative risk information and no effect of age, race or education	↔
Lipkus 2006	Combined responses to three questions about how worried, fearful and anxious they were about developing breast cancer	No difference between participants provided with either no risk information or absolute or absolute plus comparative risk information but those told that they “did not have more than the average number of risk factors” had lower combined worry, anxiety and fear at follow-up than those told that they had more than the average number (mean at follow-up adjusted for baseline 5.60 for low comparative information compared with 6.38 for high comparative information)	↔
Lipkus 2001	Combined responses to three questions about how worried, fearful and anxious they were about developing breast cancer	No difference between participants provided with absolute risk alone or absolute plus comparative risk information	↔
Holloway 2003	Individual questions includng – ‘*How anxious are you about your recent smear test*?’; ‘*How concerned are you about the chance of serious problems with your smear test in the future*?’; and ‘*How fearful are you of cervical cancer*?’	Women in intervention practices were significantly less likely to be “anxious about recent smear test” (OR: 0.81 (95%CI: 0.66 to 0.98)), “concerned about chances of serious problems with smear test in the future” (OR: 0.70 (95%CI: 0.51 to 0.95)), “fearful of cervical cancer” (OR: 0.66 (95%CI: 0.47 to 0.93))	↓

#### 3.3.2 General anxiety, depression, affect and health-related quality of life

Three studies reported general anxiety using versions of the Spielberger State Anxiety Inventory (STAI) [40]. Two RCTs showed non-statistically significant differences between women randomised to receive personalised estimates of the risk of cervical cancer during cervical screening appointments or routine care (-1.6 (95%CI: -3.5 to 0.2), p = 0.084) [23] and among 314 participants randomised to complete a self-administered decision aid for colorectal cancer (CRC) screening that included personalised information on risk of developing CRC or to receive a booklet about the Australian CRC screening guidelines [41]. The third study by van Erkelens et al. [42] measured anxiety using a Dutch version of the STAI alongside the Hospital Anxiety Depression Scale before and two weeks after 287 women had completed an online self-test that identified those at increased Familial Breast Cancer risk based on the Dutch breast cancer guidelines. It was the only study to report results separately for women at population risk and those at moderate (relative risk ≥2-3) or high risk (relative risk >4) of breast cancer. In women at population risk of breast cancer (n = 272), state-anxiety significantly decreased immediately after taking the test (mean change from baseline -2 (95% CI -2 to -1), p < 0.001) and both state anxiety and trait anxiety significantly decreased at two weeks (mean change from baseline -3 (95% CI -5 to -2) and -1 (95% CI -2 to -1) respectively, p for both ≤0.002). There was no change in distress among those participants at two weeks and no significant changes in any outcomes in the 15 women at increased familial breast cancer risk.

Affect was measured in one RCT using the Positive and Negative Affect Scale (PANAS) [43] in which female undergraduates received absolute risk feedback with or without comparative information [37]. No significant between-group differences in affect were observed. Health-related quality of life was additionally measured in two RCTs [44,45] using the SF-36 [46]. Both reported a significant increase in score on the SF-36 at follow-up in the intervention group compared with the control group.

## 4 Discussion and conclusion

### 4.1 Discussion

This study is, to our knowledge, the first comprehensive review of the impact of interventions incorporating provision of personalised cancer risk information based on non-genetic risk factors on accuracy of risk perception and psychological responses among individuals at population level risk. A particularly novel aspect is that in the synthesis we have been able to distinguish between recall of risk information and risk perception and have shown that, while immediately after provision of risk information 87% of individuals were able to recall the absolute risk estimate, less than half believed that to be their risk, with up to 71% believing their risk to be higher than the estimate. These findings in particular highlight the conceptual problems in understanding risk information and the tendency for people to resist information that is communicated to them by experts that have previously been reported across both cancer and other diseases [5]. Among these, qualitative studies have shown that risk perception is not as simple as recalling a number and that the processing of risk information is not purely ‘rational’ or ‘objective’ [47]. Instead, an individual’s perception of risk is based on a complex integration of cognitive and social biases arising from cultural, personal or lay theories of disease and risk, and past experiences, expectations and beliefs [32,34,[47], [48], [49], [50], [51], [52]]. The studies included in this review support the view that, rather than simply replacing their prior beliefs concerning their risk of developing cancer with new information, individuals appear instead to be using the new risk information to update their prior beliefs, analogous to Bayesian inference. The extent to which individuals over- or under-estimate their risk at baseline decreases after provision of risk information (reflected by an increase in accuracy) but many individuals continue to, in most cases, overestimate their risk.

The complex cognitive processes involved in this conceptualisation of risk may in part also explain our finding that risk-based inventions improve accuracy of absolute risk perception but not comparative risk. By its very nature comparative risk is a more emotive and less abstract construct [8]. It may therefore be more prone to cultural, cognitive and social biases and in turn more resistant to change. For the same reasons, however, comparative risk may sometimes play a more important role in influencing decisions concerning health behaviours.

The observed discrepancy between the risk estimate and perceived risks may also reflect varying levels of numeracy and the difficulties people often have understanding risk information [53,54]. This is supported by the finding in this review that among women with an estimated risk <1.5%, those with high numeracy had greater increases in accuracy than those with low numeracy [36]. Numerical misunderstanding was also given as a reason for feeling that their risk was higher or lower by women who recalled their risk estimate correctly but gave a different response when asked about their perceived risk in the study by Lipkus et al. [32].

The finding that individuals tend to overestimate their risk prior to receiving risk information and that provision of risk information has no effect or reduces cancer worry, anxiety and depression has also been reported for other diseases, including diabetes [55] and cardiovascular disease [56], and following communication of genetic risk [13,57]. Cancer specific worry has been reported to predict engagement in prevention initiatives [58]. This observed reduction in cancer worry, anxiety and fear may in part, therefore, explain the lack of association between provision of risk information and behaviour change [16].

These findings must however be interpreted within the limitations of this review. We performed it following accepted best practice with independent screening of full-text articles for inclusion and double data extraction and quality assessment [15]. Nevertheless, there are a number of limitations. Firstly, while we screened over 35,000 articles from four electronic databases and the reference lists of included articles, we did not specifically search for additional grey literature and were unable to assess publication bias formally. It is therefore possible that there are additional studies of relevance to this review question that we did not include. Given the number of articles screened and the high proportion of those with negative findings, however, we think it unlikely that these would change the overall findings. Secondly, the design of the included studies, definitions of accuracy of risk perception, and the range of ways in which psychological outcome measures were collected and reported varied substantially. For example, the 23 included studies incorporated 12 different measures of risk accuracy and eight of worry. This range of measures has been reported previously [12,14] and made summarising and pooling the findings difficult and meant we were only able to include a small number of the studies in the meta-analysis, limiting the strength of those results. This was further limited by many of the included studies also only presenting data for outcomes where significant changes had been observed, including only a statement of no change for other outcomes. Thirdly, risk was communicated to individuals in different formats and many of the interventions included written or verbal information alongside risk estimates. Isolating the effect of the risk information or any differences between formats was not possible. This is likely to have less of an impact on the measures of risk perception but may have influenced the psychological outcomes. Fourteen of the 23 studies also looked at breast cancer, all but two were in the US and all were at risk of potential recruitment bias. Together these limit the generalisability of the findings. Particularly for accuracy of risk perception, most of the studies only reported outcomes either immediately or a few weeks after provision of risk information. The findings therefore largely reflect the short term impact of provision of risk information.

### 4.2 Conclusion

This review shows that immediately after provision of risk information 87% of individuals were able to recall the absolute risk estimate that they had been given. However, less than half believed that to be their risk, with up to 71% believing their risk to be higher than the given estimate. Provision of risk information increased accuracy of perceived risk immediately after risk information and reduced mean perceived risk among groups who overestimated their risk at baseline. However over half of individuals remained inaccurate, with most perceiving their risk as higher than the risk estimate that they had been provided with. By comparison, there was no significant effect on comparative risk accuracy, either immediately or in the short/medium term and either no effect or a reduction in worry, anxiety or depression, with no evidence of differences with age, race, level of education or presentation of risk.

The review itself also highlights a number of important messages for researchers. These include the need for: consistent measures of risk accuracy and psychological responses to facilitate comparison across studies; sub-group analyses, particularly for psychological responses, in individuals who over-estimate or under-estimate their risk at baseline; studies including other cancer types, outside the US, and among men and people of diverse socioeconomic and cultural groups to improve the generalisability of the results; and better reporting of negative results. Attempting to measure risk perception with a single number is also unlikely to capture the complex cognitive processes involved in the conceptualisation of risk. Researchers should therefore consider using broader risk perception instruments, such as the Tripartite model of risk perception which includes assessment of susceptibility to disease (deliberative risk perception) alongside measures of the affective and experiential components of risk perception, including cancer-specific worry, anxiety and fear [59]. Not only is this model more likely to capture the range of cognitive processes, but it has been shown to predict intention to change health-related behaviour more accurately than unidimensional models of risk perception. Risk conviction, the subjective sense of certainty that one knows what one’s perceived risk is and the confidence that this risk perception is accurate [60] may also be a more sensitive measure of the impact of provision of risk information.

### 4.3 Practice implications

Perhaps the most important message from this review for clinical practice is the recognition that individuals who appear to understand and be able to recall risk information provided to them most likely do not believe that the risk information reflects their own risk. As described above, the reasons for this are complex and, as a result, are unlikely to be specific to cancer or overcome within a single consultation or by a single intervention. However, by being aware of the limits of provision of information and cognisant of the context in which each person is using the information to construct an individual perception of risk, clinicians will be better able to tailor the explanations of risk to their patients and support their understanding and shared-decision making.

## Funding

JUS is funded by a Cancer Research UK Cancer Prevention Fellowship (C55650/A21464). BS was supported by the Medical Research Council [MC_UU_12015/4]. SJS is supported by the Medical Research Council www.mrc.ac.uk [Unit Programme number MC_UU_12015/1]. The University of Cambridge has received salary support in respect of SJG from the NHS in the East of England through the Clinical Academic Reserve. The views expressed are those of the authors and not necessarily those of the NHS, Department of Health or the US NIH. All researchers were independent of the funding body and the funder had no role in data collection, analysis and interpretation of data; in the writing of the report; or decision to submit the article for publication.

## Author statement file

The contributions of authors to the manuscript are as follows: a) study concept and design: Usher-Smith, Griffin, Silarova; b) acquisition, analysis or interpretation of data: Usher-Smith, Bayne, Fairey, Silarova, Griffin, Sharp, Klein, Sutton; c) drafting of the manuscript: Usher-Smith, Bayne, Fairey; d) critical revision of the manuscript for important intellectual content: Griffin, Klein, Silarova, Sharp, Klein, Sutton; e) obtained funding: Usher-Smith, Griffin.

## Data sharing

All data are available from the reports or authors of the primary research. No additional data is available.

## Declaration of Competing Interest

None.

## References

[1] Marteau T.M., French D.P., Griffin S.J., Prevost A.T., Sutton S., Watkinson C., Attwood S., Hollands G.J. (2010). Effects of communicating DNA-based disease risk estimates on risk-reducing behaviours. Cochrane Database Syst. Rev..

[2] French D.P., Cameron E., Benton J.S., Deaton C., Harvie M. (2017). Can communicating personalised disease risk promote healthy behaviour change? A systematic review of systematic reviews. Ann. Behav. Med..

[3] Niknian M., McKinlay S.M., Rakowski W., Carleton R.A. (1989). A comparison of perceived and objective CVD risk in a general population. Am. J. Public Health.

[4] Burton H., Chowdhury S., Dent T., Hall A., Pashayan N., Pharoah P. (2013). Public health implications from COGS and potential for risk stratification and screening. Nat. Genet..

[5] Han P.K., Lehman T.C., Massett H., Lee S.J., Klein W.M.P., Freedman A.N. (2009). Conceptual problems in laypersons’ understanding of individualized cancer risk: a qualitative study. Health Expect..

[6] Reventlow S., Hvas A.C., Tulinius C. (2001). “In really great danger…” the concept of risk in general practice. Scand. J. Prim. Health Care.

[7] Lupton D. (1999). Risk.

[8] Brewer N.T., Chapman G.B., Gibbons F.X., Gerrard M., McCaul K.D., Weinstein N.D. (2007). Meta-analysis of the relationship between risk perception and health behavior: the example of vaccination. Heal. Psychol..

[9] Kinsinger S.W., McGregor B.A., Bowen D.J. (2009). Perceived Breast Cancer Risk, Social Support, and Distress Among a Community-Based Sample of Women. J. Psychosoc. Oncol..

[10] Waters E.A., Arora N.K., Klein W.M.P., Han P.K.J. (2010). Perceived risk, trust and health-related quality of life among cancer survivors. Ann. Behav. Med..

[11] van Dooren S., Rijnsburger A.J., Seynaeve C., Duivenvoorden H.J., Essink-Bot M.-L., Tilanus-Linthorst M.M.A., de Koning H.J., Tibben A. (2004). Psychological distress in women at increased risk for breast cancer: the role of risk perception. Eur. J. Cancer.

[12] Dieng M., Watts C.G., Kasparian N.A., Morton R.L., Mann G.J., Cust A.E. (2014). Improving subjective perception of personal cancer risk: systematic review and meta-analysis of educational interventions for people with cancer or at high risk of cancer. Psychooncology..

[13] Butow P.N., a Lobb E., Meiser B., Barratt A., Tucker K.M. (2003). Psychological outcomes and risk perception after genetic testing and counselling in breast cancer: a systematic review. Med. J. Aust..

[14] Smerecnik C.M.R., Mesters I., Verweij E., De Vries N.K., De Vries H. (2009). A systematic review of the impact of genetic counseling on risk perception accuracy. J. Genet. Couns..

[15] Moher D., Liberati A., Tetzlaff J., Altman D.G. (2010). Preferred reporting items for systematic reviews and meta-analyses: the PRISMA statement. Int. J. Surg..

[16] Usher-Smith J., Silarova B., Sharp S.J., Mills K., Griffin S.J. (2018). Effect of interventions incorporating personalised cancer risk information on intentions and behaviour: a systematic review and meta-analysis of randomised controlled trials. BMJ Open.

[17] Critical Appraisal Skills Programme (CASP) Checklists, (n.d.). http://www.casp-uk.net/casp-tools-checklists.

[18] Hedges L.V., Vevea J.L. (1998). Fixed- and random-effects models in meta-analysis. Psychol. Methods.

[19] Knol M.J., Le Cessie S., Algra A., Vandenbroucke J.P., Groenwold R.H.H. (2012). Overestimation of risk ratios by odds ratios in trials and cohort studies: alternatives to logistic regression. CMAJ..

[20] Haldane J.B. (1956). The estimation and significance of the logarithm of a ratio of frequencies. Ann. Hum. Genet..

[21] Timmermans D.R.M., Oudhoff J.P. (2012). Weergave van risico’s in de KWF Kanker Risico Test. Ned Tijdschr Geneeskd..

[22] Gail M.H., Brinton L.A., Byar D.P., Corle D.K., Green S.B., Schairer C., Mulvihill J.J. (1989). Projecting individualized probabilities of developing breast cancer for white females who are being examined annually. J. Natl. Cancer Inst..

[23] Holloway R.M., Wilkinson C., Peters T.J., Russell I., Cohen D., Hale J., Rogers C., Lewis H. (2003). Cluster-randomised trial of risk communication to enhance informed uptake of cervical screening. Br. J. Gen. Pract..

[24] Sherratt F.C., Marcus M.W., Robinson J., Field J.K. (2016). Utilizing lung Cancer risk prediction models to promote smoking cessation. Am. J. Health Promot..

[25] Davis S., Stewart S., Bloom J. (2004). Increasing the accuracy of perceived breast cancer risk: results from a randomized trial with cancer information service callers. Prev. Med..

[26] Helmes A.W., Culver J.O., Bowen D.J. (2006). Results of a randomized study of telephone versus in-person breast cancer risk counseling. Patient Educ. Couns..

[27] Lipkus I.M., Klein W.M.P. (2006). Effects of communicating social comparison information on risk perceptions for colorectal cancer. J. Health Commun..

[28] Livaudais-Toman J., Karliner L.S., Tice J.A., Kerlikowske K., Gregorich S., Pérez-Stable E.J., Pasick R.J., Chen A., Quinn J., Kaplan C.P. (2015). Impact of a primary care based intervention on breast cancer knowledge, risk perception and concern: a randomized, controlled trial. Breast.

[29] Quillin J.M., Fries E., McClish D., Shaw de Paredes E., Bodurtha J. (2004). Gail model risk assessment and risk perceptions. J. Behav. Med..

[30] Wang C., Sen A., Ruffin M.T., Nease D.E., Gramling R., Acheson L.S., O’Neill S.M., Rubinstein W.S. (2012). Family history assessment: impact on disease risk perceptions. Am. J. Prev. Med..

[31] Weinstein N.D., Atwood K., Puleo E., Fletcher R., Colditz G., Emmons K.M. (2004). Colon Cancer: risk perceptions and risk communication. J. Health Commun..

[32] Lipkus I., Biradavolu M., Fenn K., Keller P., Rimer B. (2001). Informing women about their breast cancer risks: truth and consequences. Health Commun..

[33] Lipkus I.M., Klein W.M.P., Rimer B.K. (2001). Communicating breast cancer risks to women using different formats. Cancer Epidemiol. Biomarkers Prev..

[34] McCaul K., Canevello A., Mathwig J., Klein W. (2003). Risk communication and worry about breast cancer. Psychol. Health Med..

[35] Emmons K., Wong M., Puleo E., Weinstein N., Fletcher R., Colditz G. (2004). Tailored computer-based cancer risk communication: correcting colorectal Cancer risk perception. J. Health Commun..

[36] Seitz H.H., Gibson L., Skubisz C., Forquer H., Mello S., Schapira M.M., Armstrong K., Cappella J.N. (2016). Effects of a risk-based online mammography intervention on accuracy of perceived risk and mammography intentions. Patient Educ. Couns..

[37] Dillard A.J., McCaul K.D., Kelso P.D., Klein W.M.P. (2006). Resisting good news: reactions to breast cancer risk communication. Heal. Commun..

[38] Lipkus I.M., Klein W.M.P., Skinner C.S., Rimer B.K. (2005). Breast cancer risk perceptions and breast cancer worry: what predicts what?. J. Risk Res..

[39] Rimer B.K., Halabi S., Sugg Skinner C., Lipkus I.M., Strigo T.S., Kaplan E.B., Samsa G.P. (2002). Effects of a mammography decision-making intervention at 12 and 24 months. Am. J. Prev. Med..

[40] Spielberger C. (1983). State-trait Anxiety Inventory for Adults.

[41] Trevena L., Irwig L., Barratt A., Trevena L.J., Irwig L., Barratt A. (2008). Randomized trial of a self-administered decision aid for colorectal cancer screening. J. Med. Screen..

[42] van Erkelens A., Sie A.S., Manders P., Visser A., Duijm L.E., Mann R.M., ten Voorde M., Kroeze H., Prins J.B., Hoogerbrugge N. (2017). Online self-test identifies women at high familial breast cancer risk in population-based breast cancer screening without inducing anxiety or distress. Eur. J. Cancer.

[43] Watson D., Clark L.A., Tellegen A. (1988). Development and validation of brief measures of positive and negative affect: the PANAS scales. J. Pers. Soc. Psychol..

[44] Bowen D.J., Powers D. (2010). Effects of a mail and telephone intervention on breast health behaviors. Health Educ. Behav..

[45] Bowen D.J., Powers D., Greenlee H. (2006). Effects of breast Cancer risk counseling for sexual minority women. Health Care Women Int..

[46] Ware J.E., Sherbourne C.D. (1992). The MOS 36-item short-form health survey (SF-36). I. Conceptual framework and item selection. Med. Care.

[47] Lipworth W.L., Davey H.M., Carter S.M., Hooker C., Hu W. (2010). Beliefs and beyond: what can we learn from qualitative studies of lay people’s understandings of cancer risk?. Health Expect..

[48] Heiniger L., Butow P.N., Charles M., Price Ma. (2015). Intuition versus cognition: a qualitative exploration of how women understand and manage their increased breast cancer risk. J. Behav. Med..

[49] Walter F.M., Emery J. (2006). Perceptions of family history across common diseases: a qualitative study in primary care. Fam. Pract..

[50] Bottorff J.L., Ratner P.A., Johnson J.L., Lovato C.Y., Joab S.A. (1998). Communicating cancer risk information: the challenges of uncertainty. Patient Educ. Couns..

[51] Kwok C., Sullivan G. (2006). Influence of traditional Chinese beliefs on cancer screening behaviour among Chinese-Australian women. J. Adv. Nurs..

[52] Hilgart J., Phelps C., Bennett P., Hood K., Brain K., Murray A. (2010). “I have always believed I was at high risk…” the role of expectation in emotional responses to the receipt of an average, moderate or high cancer genetic risk assessment result: a thematic analysis of free-text questionnaire comments. Fam. Cancer.

[53] Keller C., Siegrist M. (2009). Effect of risk communication formats on risk perception depending on numeracy. Med. Decis. Making.

[54] Lipkus I.M., Peters E. (2009). Understanding the role of numeracy in health: proposed theoretical framework and practical insights. Health (Irvine Calif).

[55] Godino J.G., van Sluijs E.M.F., Marteau T.M., Sutton S., Sharp S.J., Griffin S.J. (2016). Lifestyle advice combined with personalized estimates of genetic or phenotypic risk of type 2 diabetes, and objectively measured physical activity: a randomized controlled trial. PLoS Med..

[56] Usher-Smith J., Silarova B., Schuit E., Moons K.G.M., Griffin S.J. (2015). Impact of provision of cardiovascular disease risk estimates to healthcare professionals and patients: a systematic review. BMJ Open.

[57] Hollands G.J., French D.P., Griffin S.J., Prevost A.T., Sutton S., King S., Marteau T.M. (2016). The impact of communicating genetic risks of disease on risk-reducing health behaviour: systematic review with meta-analysis. BMJ.

[58] Hay J.L., McCaul K.D., Magnan R.E. (2006). Does worry about breast cancer predict screening behaviors? A meta-analysis of the prospective evidence. Prev. Med. (Baltim).

[59] Ferrer R.A., Klein W.M.P., Persoskie A., Avishai-Yitshak A., Sheeran P. (2016). The tripartite model of risk perception (TRIRISK): distinguishing deliberative, affective, and experiential components of perceived risk. Ann. Behav. Med..

[60] Taber J.M., Klein W.M.P. (2016). The role of conviction in personal disease risk perceptions: What can we learn from research on attitude strength?. Soc. Personal. Psychol. Compass.

