# Supplementary material for: Effect of interventions including provision of personalised cancer risk information on accuracy of risk perception and psychological responses: A systematic review and meta-analysis
Source: Patient Educ Couns. 2020 Jan;103(1):83–95. doi: 10.1016/j.pec.2019.08.010 (PMC6919334; doi:10.1016/j.pec.2019.08.010)
Supplement: Supplementary file 2 [file mmc2.pdf]

Appendix File A.2. Quality assessment of included studies

Author, date	Study addressed a clearly focused issue	Use of an appropriate method / Randomisation (for RCTs)	Recruitment / comparability of study groups at baseline	Blinding (for RCTs)	Outcome measurement	Comparability of study groups during study (for RCTs)	Follow up (for longitudinal studies)	Confounding factors (for non-RCTs):	Overall
Bowen 2006	⬤	⬤	⬤	•	⬤	⬤	⬤	n/a	H
Bowen 2010	⬤	⬤	⬤	•	⬤	⬤	⬤	n/a	H
Davis, 2004	⬤	⬤	●	•	⬤	●	⬤	n/a	M
Dillard, 2006a	●	●	•	•	⬤	•	⬤	n/a	L-M
Dillard, 2006b	●	●	•	•	⬤	•	⬤	n/a	L-M
Emmons, 2004	⬤	⬤	●	•	⬤	●	n/a	n/a	M-H
Helmes, 2006	⬤	●	●	•	⬤	●	●	n/a	M-H
Holloway, 2003	⬤	⬤	⬤	●	⬤	●	⬤	n/a	H
Lipkus , 2006	⬤	⬤	•	•	⬤	•	n/a	n/a	M
Lipkus, 2001a	⬤	⬤	•	n/a	⬤	n/a	⬤	•	M-H
Lipkus, 2001b	⬤	●	•	n/a	⬤	n/a	n/a	●	M
Lipkus, 2005	⬤	•	•	n/a	⬤	n/a	n/a	●	M
Livaudais-Toman, 2015	⬤	⬤	⬤	⬤	⬤	⬤	⬤	n/a	H
McCaul, 2003	⬤	⬤	•	•	⬤	•	⬤	n/a	M
Quillin, 2004	⬤	⬤	•	•	⬤	•	●	n/a	M
Rimer, 2002	⬤	⬤	●	•	⬤	●	⬤	n/a	M-H

Seitz, 2016	H	H	H	L	H	M	n/a	n/a	M-H
Sherrat, 2016	H	H	H	M	H	M	H	n/a	H
Timmermans 2012	M	M	M	L	H	M	n/a	n/a	M
Trevena 2008	M	M	H	H	H	M	M	n/a	M-H
van Erkelens, 2017	H	M	L	L	H	L	L	n/a	L-M
Wang, 2012	H	H	H	L	H	H	H	n/a	H
Weinstein, 2004	H	M	L	L	H	L	n/a	n/a	L-M

• Low (L) ● Medium (M) ⬤ High (H)
